# Forest Manners Exchange: Forest as a Place to Remedy Risky Behaviour of Adolescents: Mixed Methods Approach

**DOI:** 10.3390/ijerph18115725

**Published:** 2021-05-26

**Authors:** Karolina Macháčková, Roman Dudík, Jiří Zelený, Dana Kolářová, Zbyněk Vinš, Marcel Riedl

**Affiliations:** 1 Department of Forestry and Wood Economics, Czech University of Life Sciences Prague, Faculty of Forestry and Wood Sciences, Kamýcká 129, 6-Suchdol, 16500 Praha, Czech Republic; dudik@fld.czu.cz (R.D.); riedl@fld.czu.cz (M.R.); 2 Department of Hotel Management, Institute of Hospitality Management in Prague, Svídnická 506, 18200 Prague, Czech Republic; zeleny@vsh.cz (J.Z.); vins@vsh.cz (Z.V.); 3 Department of Languages, Institute of Hospitality Management in Prague, 18200 Prague, Czech Republic; kolarova@vsh.cz

**Keywords:** forest fauna community, communication, social behaviour, aggression, projective tests, Shinrin-yoku, forest pedagogy

## Abstract

This paper evaluates the impact of the forest environment on aggressive manifestations in adolescents. A remedial educative programme was performed with 68 teenagers from institutions with substitute social care with diagnoses F 30.0 (affective disorders) and F 91.0 (family-related behavioural disorders), aged 12–16 years. Adolescents observed patterns of prosocial behaviour in forest animals (wolves, wild boars, deer, bees, ants, squirrels and birds), based on the fact that processes and interactions in nature are analogous to proceedings and bonds in human society. The methodology is based on qualitative and quantitative research. Projective tests (Rorschach Test, Hand Test, Thematic Apperception Test) were used as a diagnostic tool for aggressive manifestations before and after forest therapies based on Shinrin-yoku, wilderness therapy, observational learning and forest pedagogy. Probands underwent 16 therapies lasting for two hours each. The experimental intervention has a statistically significant effect on the decreased final values relating to psychopathology, irritability, restlessness, emotional instability, egocentrism, relativity, and negativism. Forest animals demonstrated to these adolescents ways of communication, cooperation, adaptability, and care for others, i.e., characteristics without which no community can work.

## 1. Introduction

Every living organism manifests itself in relationship with other organisms in the environment. However, sometimes relationships are disrupted by aggression and social maladaptation, which is more serious when this concerns children and adolescents [1,2,3]. Every individual has aggression of varying intensity and to a different extent, and it cannot be entirely ruled out of our lives. At the same time, aggression is an adaptation mechanism that helps us survive and overcome various obstacles. [4,5]. The difference between the concepts of aggression and aggressiveness can be summarised in two areas: (1) aggressiveness is a specific trait, a character trait. It is determined biologically (heredity), cognitively (learning) and psycho-socially (emotional, along with the influence of the external environment) [6,7]. This property is within each person to a greater or lesser extent [8]. (2) Aggression is understood as any form of behaviour intended to harm someone intentionally [9,10] Aggression can be suppressed (without external expression), verbal (swearing, writing complaints), against things (destruction, tearing, breaking objects and things) and against animals and people [11,12,13]. According to the World Health Report (WHO 2010) [14], in that period, 1.6 million people lost their lives in the world due to violent behaviour. Half committed suicide, a third were murdered, and a fifth were lives lost in armed conflict.

Aggressive tendencies are created in humans in the first years of life based on hereditary dispositions, instinctive equipment, upbringing, learning, previous experience, and environmental influence [15]. Aggression must be controlled, cultivated, regulated and directed in a pro-socially effective direction so that relations between people and society’s norms are not disrupted [16].

Many young people cannot integrate properly into society, often accompanied by conflicts and personality problems, including increased aggressive behaviour [17]. Prevention of undesirable behaviour in children and adolescents is one of the necessities for achieving a certain quality of life in adulthood. In modern society, Kagan [18] identifies adolescence as the riskiest period on the path to adulthood, and it is a sensitive period for the development of risky and problematic behaviour. At the forefront of the interest in preventive and corrective activities, there is the syndrome of risky and problem behaviour, which includes truancy, bullying, extreme manifestations of aggression, racism and xenophobia, the negative effect of sects, sexually risky behaviour, and addictive behaviour [19,20].

Adolescents try to cope with the developmental tasks of the transition from childhood to adulthood through risky behaviour. Therefore, it is necessary to help them find healthy alternatives, such as remedial educative programmes, to fulfil the same function [20,21,22]. The family, peers, and the immediate surroundings of the adolescent have a significant protective role. Schools and other educational facilities have a special status, operating across the board in an interdisciplinary, long-term, and continuous manner [23].

### 1.1. Youth Behavioural Strategy

In this research, attention was focused on the manifestations of aggressive behaviour in adolescents. The standard procedure for managing aggressive manifestations is to limit external irritant stimuli, to provide space for the patient to express his/her feelings, or pharmacological intervention. The recommended methods to manage aggression include creative activity, physical activity and relaxation exercises. Aggression can also be mastered by direct experience or observation of other people’s social interactions, i.e., observational learning [24,25,26]. The learning model could be family members, people from the immediate circle, symbolic models from the mass media and virtual reality, or computer games with violent and brutal themes.

An emerging treatment that utilises wilderness therapy to help adolescents struggling with behavioural and emotional problems is considered here, i.e., outdoor behavioural healthcare (OBH) [27]. Wilderness treatment is one option of care that effectively treats children and adolescents presenting aggressive behaviour [28]. Wilderness therapy combines traditional therapy techniques with group therapy in a wilderness setting, approached with therapeutic intent [29]. Adolescents demonstrated marked improvements in the following areas: anxiety and depression, substance abuse and dependency, disruptive behaviour, defiance and opposition, impulsivity, suicidality, violence, sleep disruption, school performance, and interpersonal relationships. Russel [29] conducted a study lasting for 45 days. Adolescent client well-being was evaluated using the Youth Outcome Questionnaire (Y-OQ) and the Self Report-Youth Outcome Questionnaire (SR Y-OQ) [30]. Complete data sets at admission and discharge were collected for 523 client self-reports and 372 parent assessments. Results indicated that, at admission, clients exhibited symptoms similar to inpatient samples, which were, on average, significantly reduced at discharge. Outcomes had been maintained at 12-months post-treatment. [30,31]. Russel [31] evaluated youth well-being 24-months after the conclusion of outdoor behavioural healthcare (OBH) treatment and explored youth transition to various post-treatment settings. The results suggest that 80% of parents and 95% of youths perceived OBH treatment as practical, the majority of clients were doing well at school, and family communication had improved. Aftercare was utilised by 85% of the youths and was perceived as a crucial component in facilitating the transition from an intensive wilderness experience to family, peer and school environments.

Other studies also show that participants who had walked in nature reported less anger and more positive emotions than those who engaged in other activities (walking in an urban area, sitting quietly while reading magazines or listening to music) [32,33,34,35]. The time spent in nature improves physiological relaxation and the immune function recovery response [36,37]. Nature therapy can provide emotional healing, decrease blood pressure, improve a person’s general sleep-wake cycle, improve relationship skills, reduce stress, and reduce aggression [38,39]. Research studies included in the Stanford analysis [40] have also found that the natural environment develops potential, and participants gain both tacit knowledge and necessary depth of knowledge.

### 1.2. Study Design Based on Forest Programme

Prosocial behaviour is a successful strategy used by human and non-human individuals living in stable, long-lasting social groups [41,42]. The human’s limbic system allows us to experience joy, sadness, fear, and pleasure; these brain structures are common to humans, mammals, birds, and fish at a significantly lower evolutionary stage [43]. This finding is followed by several studies dealing with animal emotions [44,45,46]. The authors concluded that animals show affection, care for their species, can mourn and face danger together. Another point of view is provided by Pavlík and Kopčaj [47,48,49], claiming that processes and interactions in nature are analogous to human social interactions. Rösler [50] introduces the term “Nature Ideas Exchange”: nature offers many analogies applicable to practice, via which the principles of prosocial, positive emotional behaviour and cooperation can be presented uniquely, as caring for others, coexistence, cooperation, adaptation, and innovation occur every day in nature. The constant pressure towards assimilation has led to high-level specialisations, sophisticated survival strategies, and collaborative models [50].

We, therefore, assumed that this could work the other way around: if young people with risky behaviour could not acquire prosocial behaviour patterns such as care, fidelity, compassion, cooperation and adaptation in their families, this tacit knowledge could be gained by observation in nature of forest fauna examples. In the forest environment, the effects of therapy on reducing aggression can be combined with observational learning.

### 1.3. The Aim of the Research

Eisikovits [51] states that residential education should include the best possible education methods, re-education, and psychotherapy. Without these, alternative institutional care would merely consist of isolation of the child from society. Therefore, an unorthodox remedial educative programme was performed for adolescents from institutions offering substitute social care, suffering mental problems with severe risky behaviour (abuse, criminality, truancy, aggression, early sex life). The established diagnoses of adolescents are given in chapter 2.1. The research aimed to verify the transformation in adolescents’ attitudes and behaviour based on forest therapy focused on forest animals’ emotional lives. This research aimed to identify whether probands’ teamwork and social adaptation would increase. The following question has guided the study: is it possible to reduce aggressive behaviour with forest therapies based on observing the social behaviour of forest animals with the simultaneous therapeutic action of Shinrin-yoku and Outdoor Behavioural Therapy (OBH)?

The following hypothesis was established: Experimental intervention in the form of forest therapy in groups “A” and “B” will reduce manifestations of aggression compared to the original values measured before forest therapy.

## 2. Materials and Methods

Although the idea of OBH and wilderness therapies is remarkable and the study by Russell [29,30,31] yields positive results, he admits that OBH and wilderness therapy’s effectiveness reveals a consistent lack of theoretical basis, methodological shortcomings and problematic results difficult to replicate [31]. Therefore, we have focused on the primary tool for influencing attitudes and behaviour: remedial educative programmes [52] based on Shinrin-yoku, observational learning and forest pedagogy methods. Standardised psycho-diagnostic instruments (projective tests) were used to evaluate adolescents’ aggressiveness before and after taking part in the remedial educative programme.

### 2.1. Forest Therapy and Shinrin-Yoku

Forest therapy is a scientifically based method, the results of which are confirmed by independent research. Its foundations are based on the Japanese Shinrin-yoku technique [53,54]. It has been reported that forest environments have beneficial effects on human health: increased natural killer (N.K.) cell activity and in the number of N.K. cells; increase in intracellular anti-cancer proteins; blood pressure lowering and decrease in stress hormones (urinary adrenaline, noradrenaline, salivary cortisol). Up-to-date studies show that, after a few hours in the forest, the level of stress hormones falls sharply, and the immune system’s activity increases. The parasympathetic nervous system is activated due to phytoncides-chemicals released into the air mainly by conifers, auditory stimuli of wild birds singing, and visual stimuli of sunlight shining through the leaves [55,56,57,58,59,60,61]. In the Profile of Mood States (POMS) test, forest environments reduce anxiety, depression, anger, fatigue, confusion and increase the score for vigour [53], and especially for anxiety [62]. It is a therapeutic method that can be used to prevent the treatment and rehabilitation of stress disorders and civilisation diseases and help treat mental disorders such as anxiety-depressive disorders [53]. Kotera and Fido [63,64] demonstrated that the mean scores for mental well-being, self-compassion, common humanity, and mindfulness had increased significantly from pre-retreat to post-retreat.

### 2.2. Forest Pedagogy

Forest pedagogy is forest-related environmental education [65] based on the experiential method, using senses, i.e., based on experiences and feelings. According to Pestalozzi’s concept of learning with head, heart, and hand, forest pedagogy’s basic principle is the perception of nature by all senses [66]. Understanding ethical values emerge through the perception of situations, nature, and other people. Forest pedagogy develops emotional intelligence, supporting cooperation and teamwork, self-awareness and co-responsibility. The programme is carried out in groups; the individual is part of the group and is constantly exposed to many social stimuli. Forest pedagogy deals with methods such as interview, brainstorming, brainwriting, discussion, demonstration, practical activities, thematic games, competitions, simulation, situational methods and dramatisation, buzz groups, incident resolution method, maze method, aquarium method, role-playing, staging, dramatisation, themed games, and competitions [65,67,68,69]. The following activities were selected from the PAWS textbook for teenagers: name tag and symbol; animal rights; eco-audit; desert island.

### 2.3. Projective Tests as a Diagnostic Tool for Aggressive Manifestations

Projective tests are used to diagnose aggressive tendencies [6]. Doctors use these tests to determine patients’ aggressive potential as part of a psychological examination. Projective methods show implicit personality motives, and their results are challenging to distort by intentional dissimulation. We focused on three projective methods, which have been standardised as administration, evaluation, and interpretation: the Rorschach test, the Hand test, and the Thematic Apperception Test, used to examine patients with risky behaviour [70,71,72].

Projective techniques confront the tested person with an indefinite situation, to which he/she will subjectively react according to the interpretation of its meaning. The ambiguity of the situation can reveal latent personality components that the individual is unaware of [73]. Projective tests show the examined person’s psychological presentation and relation to the surrounding world by confronting the individual with a stimulating situation. Individuals perceive and interpret the tested material to reflect their psychological functioning because they project onto the situation their thinking processes, emotions, needs, anxieties, and intrapsychic conflicts [6,73].

#### 2.3.1. The Hand Test

The Hand test is a projective technique that should predict aggressive behaviour and map interpersonal tendencies [70,74,75,76]. A hand symbol is an essential tool of social contact and a means of controlling the material world; the hand plays a vital role in body language and can be a source of pleasure and pain [72]. The Hand test consists of nine cards on which the shape of a human hand is drawn, and the tenth card is empty. The respondent should tell what emotion the picture expresses: “What can this hand do”? Whether the answers express affection, communication, exhibition, acquisition, failure, reciprocity, dependence, showing off, directive, aggression, activity, passivity, fear or tension is assessed [77]. These categories are grouped into: (1) interpersonal responses related to other people; (2) environmental responses related to the impersonal area and the impersonal environment; (3) maladaptive responses, including formulations exhibiting weakness, external inhibition, failure to meet needs; (4) answers expressing distance. In this research, the Hand test was administered, evaluated, and interpreted according to Lečbych [78].

#### 2.3.2. The Rorschach Test (ROR)

The test consists of ten differently coloured boards: black and white, colourful (dark + red), and coloured boards. The purpose of the test is to determine the projection of thought processes and personality traits on indefinite objects, which have the form of ink stains of various colours and shapes [72,79,80,81,82,83]. After presenting the board, the tested person is asked the question, “What could it be, what is it like?” From the ROR answers, it is possible to deduce several variables: emotion, aggressive tendencies, personality, interpersonal relationships, the subject’s relationship to himself, and cognitive functions. The IQ band can be approximately estimated from the test.

Aggression can be assessed in ROR according to (1) how the stain is interpreted in terms of its integrity, (2) according to the determinant’s experience, (3) according to the content of interpretation of humans, animals, and other objects and (4) according to the occurrence of standard and original answers [84]. For example, the red parts of the card are often perceived as blood; their reaction will indicate how the test taker manages the feelings associated with anger or physical harm. The dark and shadowy parts of the card can cause a situation in which the test taker feels depressed. Pure colour interpretations are an indicator of affective and extroverted focus. Hue answers indicate the dominance of the intellect over emotions and affective adaptability. Chiaroscuro responses indicate moods (mostly dysphoric), suggesting a lack of tendency to control moods and an inability to control dysphoric reactions. The interpretation of stain shows the intensity and the importance of social bonds for the patient. Interpretations of animals are the most frequent content category, and if this value increases, it indicates stereotypes, unproductivity of thinking, and cumbersomeness or rigidity of thinking.

The answers were evaluated according to the frequency of banal (usual) and original responses. Banal responses occur in 30% of healthy population protocols. They are considered an indicator of intellectual contact and social adaptation of thinking. A higher incidence of these responses may also indicate rigidity or anxious–depressive syndrome.

Administration and scoring in clinical practice were implemented according to Exner’s Comprehensive System. Individual scores were evaluated based on a quantitative basis, thus allowing statistical verification [85,86]. A structured procedure characterises the Exner system for signing and interpreting results. It is a sophisticated system based on detailed research, extending the perceptual-cognitive basis of the Rorschach method, which affects diverse aspects of the responses (Exner and Harsa).

#### 2.3.3. The Thematic Apperception Test (TAT)

The TAT involves a series of picture cards depicting various ambiguous characters, scenes, and situations. Respondents must come up with a story for each of them. TAT is suitable for assessing or capturing the degree of interpersonal conflict and behavioural disorder [87,88,89]. The test consists of 31 pictures (boards) on which people (women, men, children) are drawn in many different life situations. One picture (number 16) consists of only a white area. Here, the subject has the task of projecting an idea of his arbitrary image and story: how the story goes and how it ends, what individuals think and feel, why the situation occurred, and the story’s resolution.

It is assumed that the examined person identifies with the characters and interprets the situation depending on his/her experience and conscious and unconscious needs [90,91,92].

### 2.4. Focus Groups

A focus group was interviewed to obtain the most valuable data from respondents through their mutual interaction [93], using group dynamics. Interaction structures reflected the division of social status (superiority, equivalence, subordination) and relationships between communicators (friendship, indifference, antagonism). The focus of the discussion was on the self-regulation of behaviour, cognition of emotions, and attention. The focus group sessions lasted 45 min, and 12 persons participated in each group. The group members were acquainted with the rules (all interviews take place in the forum, all members present can participate in the discussion, everyone has the right to express their opinion, everyone has the right to refuse an answer). There was an effort made for only one participant to speak in the group at a time, i.e., so that participants could take turns in expressing their opinions. For critical statements, transcription was performed (including substandard expressions).

The probands were asked the following questions:

1. Do you think that the social bonds of animals are transferable to the human world?
2. Which animal trait is the least transferable to the human world?
3. Does any animal community remind you of a similar bond in your family? (For example, the dominant father, like the wolf, is the pack leader.)
4. Which animal community do you like the most and why?
5. In which animal community would you like to be a cub?
6. In which community do you see the most substantial internal ties? Where does a pack member deploy their own life for the sake of their species? Is there a stronger bond between a wolf and a wolf cub, a female wild boar and a piglet, or a roe deer and a fawn?
7. Which animal community offers the best living conditions in packing status, access to food, safety?
8. Which community is the smartest?
9. Which warning system do you consider the most perfect, and which is the most altruistic?

The Focus Group aimed to discover attitudes towards the given issues and the most critical aspects influencing the answers. The moderator (one of the researchers) encouraged the probands to explain their points of view.

### 2.5. The Research Group

In the research group were adolescents from three institutions offering substitute social care with severe behavioural disorders, for which institutional treatment was ordered. Probands were selected by a specialist in psychiatry and a psychologist based on their documentation and anamnestic data, from which it was realistic to assume aggressive behaviour. The research group consisted of 68 probands (43 boys and 25 girls) 12–16 years old (average age was 14.8 years) along with (1) adolescents from the diagnostic range F 30.0—mood disorders, affective disorders (*n* = 37); (2) adolescents from diagnostic range F 91.0—behavioural disorders related to family relationships (*n* = 31) [94]. The entry criterion was aggressive behaviour or tendency (manifest, latent, auto-aggressive, hetero-aggressive). The research group also consisted of: (3) a control group of 1st-year high school students with a medical focus, without diagnosed behavioural disorders, aged 15 years (*n* = 34). In the control group, the presence of a mental disorder was the exclusion criterion. The control group was chosen to obtain a homogeneous group and because it was simple to motivate medical school students for a voluntary psychological examination. In all volunteers from the control group, the attending physician required a psychological examination focusing on aggression assessment. Members of the control group participated only in psychological testing; they did not undergo forest therapy.

### 2.6. The Research Course, Data Collection and Research Instruments

We used the therapeutic effect of Shinrin-yoku therapy, which was supplemented with forest pedagogy and observational learning focusing on principles of adaptation, partnership, and cooperation in forest animals. Forest therapies took place twice a week for two months (January–February 2020, two hours of therapy), and probands underwent 16 therapies lasting for two hours each in the forest, accompanied by a forest pedagogue (forester, graduate of a certified course in forest pedagogy), warden, and one of the authors of this paper. Adolescents were under the constant supervision of experienced mental health professionals (wardens), who could immediately intervene if any aggressive outburst occurred. The maximum group size was eight people according to the internal guidelines of the institution.

First, the forest pedagogue explained how individual animals in the forest behave and their specifics. He used demonstration and showed strategic partnerships, cooperation, adaptation, care, loyalty, courage and care using living bees, ants, deer, wild boars, squirrels, wolves, and birds. Probands worked in teams, searching for parallels between the forest ecosystem’s commonality and human society through observation, discussion, brainstorming, mind maps, educational games, and experiences using forest pedagogy methods [65,67,68,69].

Probands were examined with a battery of selected standardised diagnostic methods. The test battery consisted of three standardised projective tests described above. The testing was part of a comprehensive psychological examination, which also included personality questionnaires and inventories. Testing was performed in the following order: The Hand Test, 10 min, Rorschach Test (ROR), 20 min, and Thematic Apperception Test (TAT), 20 min. For TAT, it is also permissible to select a certain number of boards (for example, 10) suitable for a certain problem area, and these are exposed during the examination. We also chose this variant and selected boards for which a higher incidence of aggressive tendencies or responses was assumed. Selected boards were included: 3 BM, 7 BM, 8 BM, 14, 15, 16 (blank board), 17 GF, 18, BM, 18 GF, 20. The boards were chosen to reflect the patient’s characteristics and feelings such as suicidality, aggression, depression, feelings about death, fear, tension, and relationships with parental or important authorities. On the 16th freeboard, the patient had to create the scene and describe the story in it.

Parametricity was detected by the Shapiro-Wilk test (α = 5%) in both groups for all questions. The measured values indicated a “continuous variable” character, which corresponds to the descriptive statistic. Where parametricity was detected, a *T*-test for dependent samples was used (α = 5%), and where parametricity was not detected, the Wilcoxon test (α = 5%) was used [95]. The state of the values before the experimental intervention and after the experimental intervention were compared. Therefore, two tests comparing dependent samples were used because an experimental intervention took place in each examined case.

## 3. Results

### 3.1. Parallels found between Prosocial Behaviour of Forest Animals and Human Society

In the example of bee and plant, where bees collect pollen and guarantee the flower’s reproduction, the probands understood the essence of mutually beneficial cooperation. Probands discovered the interconnected and functional system of teamwork and care for others, in which everyone has a role and place and is willing to accept a part in the community of beehive and anthill, where the workload of each member of the community is given (some are responsible for protection and safety; others care for larvae; others forage). Based on the wolf pack and deer community (*Canis lupus, Cervidae*), probands understood that those who can learn from the older generation could expect longer life; and how teamwork and solidarity work in real-time: the pack or tribe must be collaborative and pull together. Conformity, tolerance and the necessity to adapt to the environment was conceived via the swarm and meadow grasshopper (*Conocephalus discolour*): a flock of animals behaves and moves like one living organism with a certain logic, without anyone commanding them. Insects blend in colour with their surroundings or mimic the appearance of wasps, bees, and bumblebees. Probands detected the principle of beneficial exchange based on ants (*Lasius niger*), termites (*Isoptera*) and aphids (*Aphidoidea*). A warning signal of the common jay (*Garrulus glandarius*) and great tit (*Parus major*) to which other animals responded by hiding in safety was understood as worrying about others. Adolescents summarised that altruism increases in value when we must consciously and actively deny something to help someone else, e.g., the great tit, who warned others, found herself in danger because she drew attention to the predator. The fact that energy invested into social cohesion creates remarkably resilient communities to external threats was perceived in the examples of communities of wild boars (*Sus scrofa*). After detecting that that common squirrel (*Sciurus vulgaris*) takes foreign orphaned cubs into their care, adolescents stated that there are more variations of maternal love, and whether maternal feelings arise from a subconscious stimulus or originate in conscious thinking is not critical to its quality. Probands discovered partnerships based on exchange, liaison and tolerance between wolves (*Canis lupus*) and ravens (*Corvus frugilegus*). They realised that there is nothing wrong with disparate relationships between predator and blackbirds.

Examples from forest fauna were intentionally described simply, adapted to the age and mental skills of the probands.

### 3.2. Evaluation of the Rorschach Test

The findings presented in this section result from psycho-diagnostic evaluations in 68 probands and a control group, examined by a battery of three projective tests before and after forest therapy. These results emerge from individual measurements for individual tests.

Group A includes probands with diagnosis F 30.0. affective disorders, 37 persons.

Group B includes probands with diagnosis F 91.0. family-related behavioural disorders, 31 persons. Table 1 presents group averages of ROR test before and after forest therapy.

The results show that the experimental intervention has a statistically significant effect (*p* < 0.01) on the achieved final values without exception. The experimental intervention reduces values in both groups A, B, and all four tests (colours, animals, blood, reality).

**Figure 1:**
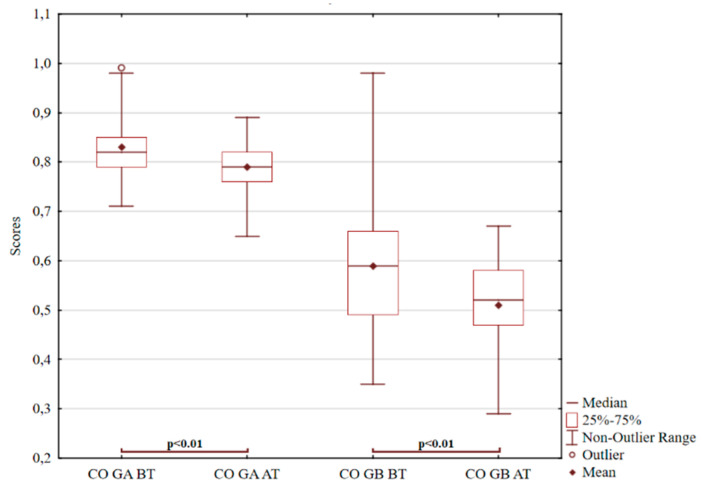
ROR for pure colour answers. Notes for Graph 1–6: BT before treatment; AT after treatment; GA Group A; GB Group B; CO pure colour answers; AN interpretation of animal; BL interpretation of blood; RE contact with reality.

There is a noticeable decrease in both the first and second pair, as shown in Figure 1. In group B, forest therapies reduced values previously appearing in the upper quartile and increased lower quartile values (*p* < 0.01; t = 4.75). This fact is reflected by a significantly lowered standard deviation for group B. For the A group, the outlier value was eliminated by the treatment (*p* < 0.01; z = 4.62). From the first round of ROR testing results, it is clear that adolescents with diagnosed mood disorders and affective disorders had the most significant difficulty managing impulsive tendencies. In their answers, purely coloured answers were repeated, representing impulsivity, which was harder to control and adapt to social norms. This indicates reduced rational control, where the instinctive component predominates, i.e., the tendency to discharge effects and unbraked emotionality. The unbalanced affective component dominated, which mainly shows the uncontrollable anxiety effect.

**Figure 2:**
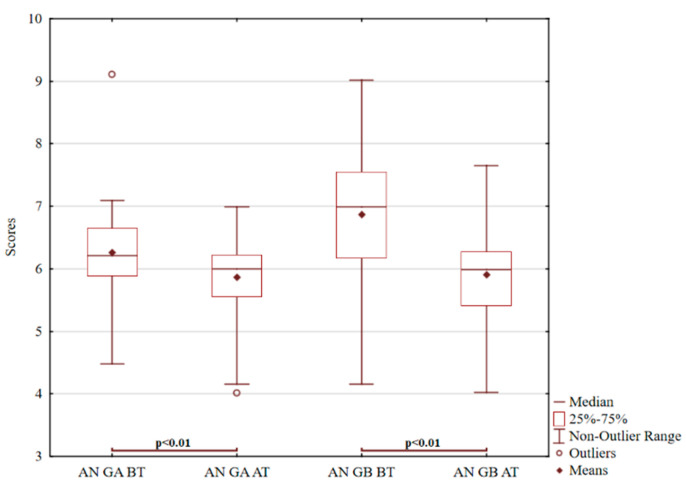
ROR for animal interpretation.

In probands with a family-related behavioural disorder, animal interpretation responses appeared most frequently, as shown in Figure 2. If the animal responses exceed the norm (30%), they are often associated with the depressive syndrome related to thinking inflexibility, stereotypes, or monotony of association processes [6]. There is a noticeable decrease in both the first and second pair. In the second pair, the positive effect is even higher because the value decrease is higher than in the first pair. There is also a significant reduction in the group’s standard deviation in the second pair that became more constant (*p* < 0.01; z = 4.80). For group A, an outlier previously appearing as a very high value was reduced by the experimental intervention. Instead, a new outlier appeared, representing the low value of evaluation (*p* < 0.01; t = 4.27).

**Figure 3:**
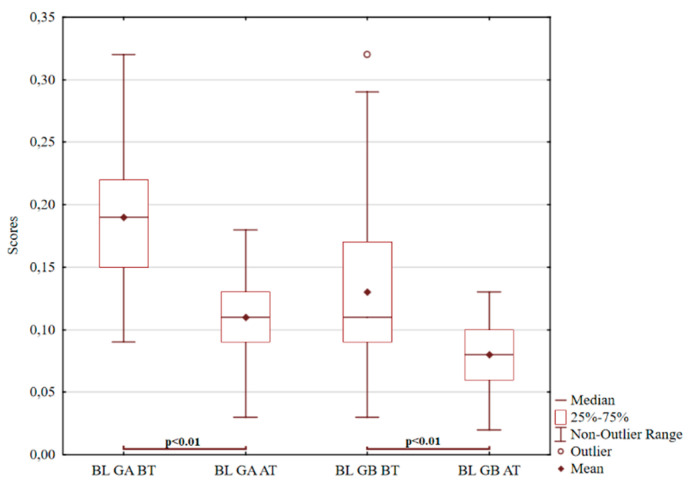
Graf 3 ROR for the interpretation of blood.

Another item tested was blood interpretation, as demonstrated in Figure 3. Responses involving blood interpretations are often associated with an excess of intrapsychic tension, acute distress, and anxiety. Various irritating stimuli from the surroundings quickly activate aggressive impulses [96,97,98,99,100,101,102]. There is a noticeable decrease in both the first and second pair (*p* < 0.01; t = 8.44). The decrease in value fluctuations is evident in both groups, but even more constantly in group B (*p* < 0.01; z = 4.11).

**Figure 4:**
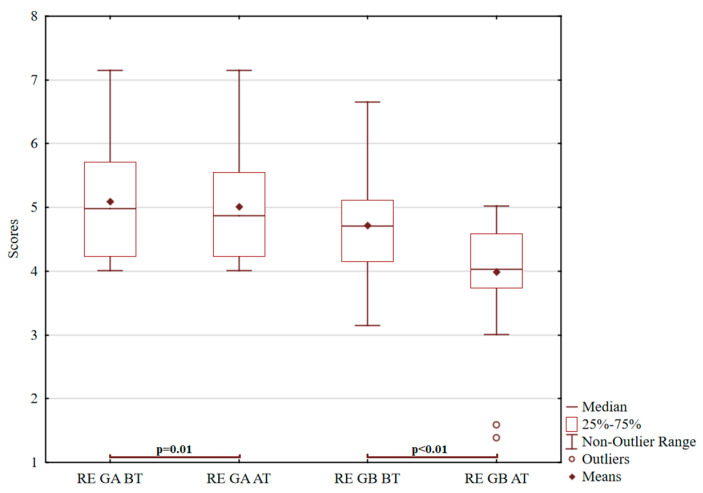
Graf 4 ROR for contact with reality.

The last item tested was contact with reality, according to Figure 4. Statistically significant difference was observed in group B (*p* < 0.01; z = 3.62), but not in group A (*p* = 0.01; t = 2.19) in the control of reality, which is an essential mediator between needs and motives in the subject’s interactions with the external environment. The range of the real estate index is in the range of 1–8 points. The norm of the real estate index is 5–7 points. Weakening (significantly below 4 points) is characteristic of more severe mental disorders. Weakness can be found in psychoses, severe psychopathies, cognitive deficits or mental retardation, i.e., for those who “do not share the world with others” but have their world of fantasy, irrationality, or autistic thoughts. An increase significantly above 7 points is also pathological. Increased control of reality may, for example, be due to a more severe depressive disorder with a predominance of stereotypy and rigidity in thinking or other neurotic features, e.g., incredibly obsessive. Depressive and the lowest psychotic probands had the highest average values on this scale. After forest therapy, there was a positive increase in the indicator of the degree of adaptation of thinking to social reality, which is indicated by common answers [103] and corresponds to the usual social rules of society, usually a guarantee of conformity to the environment necessary for human integration.

### 3.3. Evaluation of the Hand Test

From the Hand Test results (presented in Table 2), we noticed a higher frequency of responses before forest therapy, including reactions expressing interpersonal distance and an increased degree of psychopathology. Responses expressing distance express non-adaptive forms of behaviour increased feelings of stress, weakness, or avoidance of interpersonal or environmental contacts. These answers are characterised by weakened contact with reality. A high score is always pathological and reflects problems [6]. After the second round of testing in the acting out ratio (AOR) index, a decrease in bullying, threats, destruction of property, lying, escapes, racial discrimination, spraying, abuse of others, intolerance, and netholism was found. This index is considered to be a valid indicator of predicted aggression [6,104,105].

**Figure 5:**
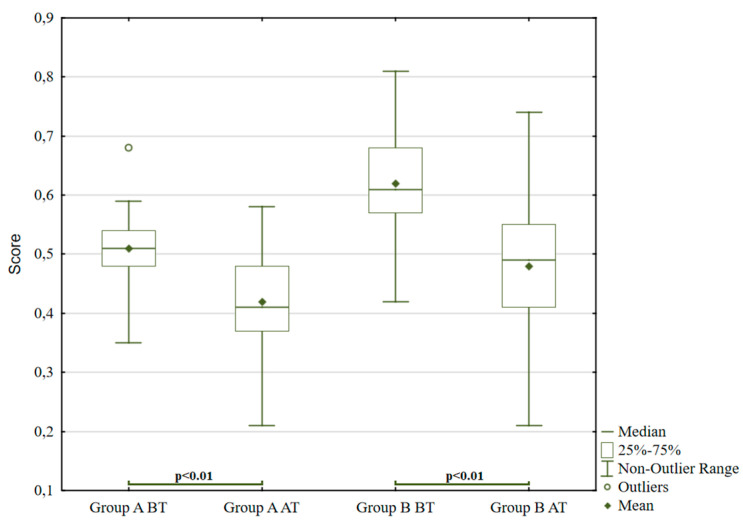
The Hand Test for non-adaptive behaviour.

**Figure 6:**
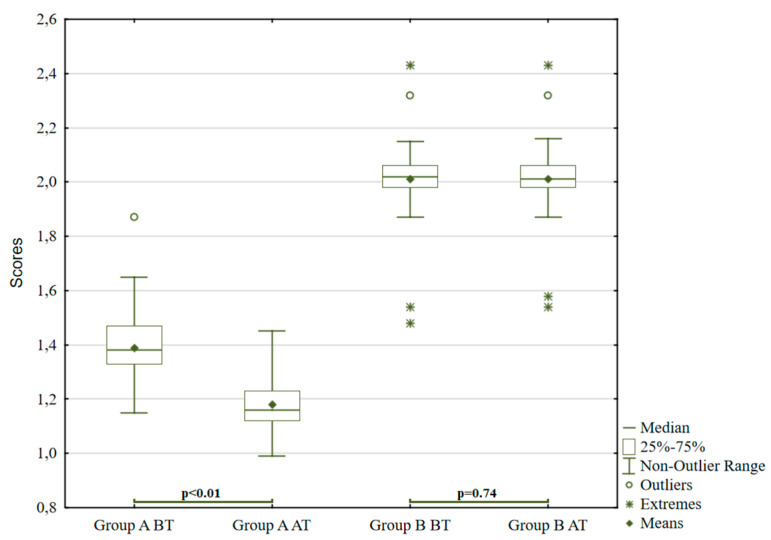
The Hand Test for pathological behavioural phenomena.

The experimental intervention has a statistically significant effect on the achieved final values, except for pathological manifestations in group B (*p* = 0.74). Statistically significant differences (*p* < 0.01) were observed in group A in values for non-adaptive behaviour and pathological manifestations (t = 6.32; t = 7.12), and in group B in values for non-adaptive behaviour (t = 5.38). There is no statistically significant decrease in values in pathological manifestations (*p* = 0.74) in group B. This fact is graphically illustrated in Figure 5. Figure 6 shows that, in the first pair, there is a noticeable decrease, but in the second pair no significant change; there are specific pathological phenomena in group B—in this group, some probands are significantly above and extremely below otherwise average values; even in this group, there are deviant individuals.

### 3.4. Evaluation of the Thematic Apperception Test

To evaluate the Thematic Apperception Test, we decided to use an interpretation system before scoring. It can be concluded that in probands with personality disorders and propensity to depression, the frequency of responses decreased by 27% and hetero-aggressive tendency by 18%.

### 3.5. Evaluation of the Hypothesis

Based on the evaluation of projective tests by statistical methods, it is possible to confirm that the experimental intervention in the form of forest therapy combined with observational learning in groups “A” and “B” led to reduced aggressive behaviour.

### 3.6. Evaluation of Focus Groups

Results of focus groups discovered the attitudes to the given issues and the most critical aspects impacting newly identified knowledge. The alternation of agents’ statements indicates group dynamics. Individual agents are marked with the abbreviation A + No.

(A32) “I understood that human babies and animal pups need the education to master the rules in adulthood.”
(A5) “I felt sorry that squirrels also take care of foreign cubs when my mother did not take care of me.”
(A18) “I understood it in mimicry; who wants to survive must adapt to the environment.”
(A17) “I liked how wolves could make friends with ravens; it is an entirely different species.”
(A9) “I thought nature was cruel, and only the predator won. I did not expect animals to help each other. I always thought that in nature, it works who with whom.”
(A21) “I was surprised that the bonds between the animals are similar to family life.”
(A9) “It seemed strange to me to compare us to animals, but finally, I enjoyed it.”
(A3) “Even in animals, there are inferior members of the tribe who get to eat only at the end, which is similar to humans; when an individual is weak, he will never be the pack leader.”
(A2) “I liked that even a tiny weak individual (tit) can protect species from a predator, which can be much larger and more robust.”

Probands discovered some parallels between their own and forest animals’ behaviour. For example, when a warden approaches, one warns the others. If they are caught in the act, the youngest weakest individual is sacrificed because he/she receives the lowest punishment. In case of damage, they try to camouflage it like that found in nature. They can also come together as a pack and speak with one voice when needed. The crucial outcome of focus groups is the finding that probands positively evaluated the compassionate manifestations of animals and reported advantages, such as reduction of anxiety.

## 4. Discussion

Institutions providing substitutional social care are not a frequent place of research. They are closed communities and are therefore demanding to obtain data. What is happening inside is not sufficiently researched. This research had the ambition to understand the social reality better examined, even if only to a small extent, given its modest scope.

### 4.1. Evaluation of Projective Tests

TAT is more associated with social adjustment, and ROR more associated with thought disorders. Both methods describe the personality in its entirety and can be used to assess instinctual component, emotions, complexes or repressed tendencies. In the first round of testing before forest therapy, a more significant increase in average values was found for responses related to emotional instability and less controlled impulses in responses involving animals, blood, and social reality checks. These interpretations are very often associated with an excess of intrapsychic tension, acute distress and anxiety. Against the background of tension, there are often insufficiently assimilated and integrated aggressive impulses, easily activated by various irritating stimuli from the environment. Comparing these research results with the conclusions of Gacono and Meloy [106], similar results were obtained in the monitored traits (colour and chiaroscuro responses). This suggests the good validity of this projective test, especially when assessing the affective component.

Similarly, in his research, Morávek [107] found statistically significant values (0.1%) in the number of aggressive responses. These were primarily responses containing offensive animals or humans. Interestingly, the Zw responses (interpretations of white spaces and interfaces that can be considered a sign of an aggressive or negative attitude) proved to be statistically insignificant, despite the literature and practice experience. In this research, similar outcomes were found.

In the projective Hand Test, statistically significant differences between groups for some items, including non-adaptive behaviour and pathological manifestations, were observed. These categories were associated with an increased incidence of psychopathological features and weakened contact with reality. No significant values were found in the predictor of aggression (AOR) or AGG (number of aggressive responses). The aggressiveness index expresses the relative weight of socially positive cooperative attitudes compared to directives and aggressiveness. Some authors, such as Klicperová [108], Morávek [107], Volkova [109] and McGill [110], consider that this index is an accurate evaluation of predicted aggression. In her research, Klicperová [108] focused on two monitored traits, AGG-aggressiveness and CRIP-damage. She evaluated the answers containing the monitored features on a three-point scale. So-called “percentage of aggression from the obtained gross scores”, which had a significant value, was calculated. In his research, Morávek [107] also focused on selected traits (AGG, number of aggressive responses; CRIP, responses involving damage to the object; and DIR, responses involving directive direction, regulation or control of others) and found statistically significant values on the scale of aggression. Probands on the AOR or AGG scale did not achieve statistically significant differences due to many factors, such as calming of the situation. Besides, the manifestations of aggression change over time and circumstances.

In the Thematic Apperception Test (TAT), statistically significant differences between the groups when assessing hetero-aggressive, auto-aggressive tendencies or topics were not observed. Responses in which hetero-aggressive tendencies predominated were most common in psychotic probands. Auto-aggressive tendencies predominated in probands with personality disorders and depressed probands. However, activated defence mechanisms that change the assessed patients’ experience and behaviour play a role, significantly suppressing aggressive tendencies.

### 4.2. Evaluation of Forest Therapy

Forest therapy has been modified with elements from wilderness therapy and OBH, Shinrin-yoku and forest pedagogy. We must distinguish between wilderness therapy and wilderness experience programmes, boot camps similar to military recruit training in a wilderness environment [111]. Actual wilderness therapy occurs under the supervision of a licensed mental health professional (psychologist, psychiatrist) who works with participants and can provide individualised treatment plans regularly monitored and evaluated. Due to the physical, cognitive, and social demands of wilderness therapy, this form of treatment may not be effective with older adults, young children, or people with specific physical disabilities. The approach may also be ineffective or unsafe for people experiencing severe or chronic mental health issues such as dementia, schizophrenia, and other similar conditions.

Due to these limitations, OBH was combined with Forest therapy based on the Shinrin-yoku methodology, observational learning and forest pedagogy. Our outcomes correspond to scientific findings by other authors [55,56,57,58,59,60,61] concerning anxiety, depression, anger decrease, and mental well-being. In this research, the positive influence of the forest environment on the development of potential acquisition of tacit knowledge was confirmed. Contemporary studies of Shinrin-yoku present fewer participants’ results than this research (12 male university students, 14 adolescent sexual offenders, 22 participants, 25 Japanese students, 27 girls aged 12 to 14 years) [56,57,62]. Furuyashi [57] conducted a more extensive study with 155 participants classified into two groups: those with and without depressive tendencies. Shinrin-yoku therapy’s length also varied: three-day retreat [64], six times a day for 15 min [55]. Before and after forest therapies, reduced items related to psychopathology, irritability, restlessness, emotional instability, egocentrism, relativity, and negativism were evaluated.

### 4.3. Limitations and Possible Follow-Up Research

This research has potential limitations relating to the application of projective tests application and the number of probands due to the institutes’ capacity. The question is whether the Rorschach test reveals the patient’s psychological centre and the process of their thoughts because probands can censor their thoughts before utterance. The evaluation can also be skewed by the evaluator’s personality, classifying the patient’s answers into predetermined categories. All three tests place significant demands on the experience and correct interpretation of the person testing the probands, i.e., the evaluator (psychologist or psychiatrist). The tests are sensitive to the immediate mental state of the subject, so a whole battery of tests is carried out, which is evaluated by an experienced doctor-psychiatrist or psychologist, who has the anamnestic data of patients in order to assess progress. However, selected projective tests seem to be a suitable tool for predicting aggressive behaviour. These methods can be recommended if trained personnel are used in a broader test battery, mainly due to the test’s high reliability and validity (especially for detecting aggressive behaviour). Therefore, based on a single projective test, there should be no clear conclusions or even a diagnosis (for example, the test subject’s immediate mental state). Projective tests should be compared with other methods (observation, questioning, objective personality tests, etc.).

Focus groups also generate certain limits. Focus group participants can censor responses according to their demands; they can respond differently under the weight of these circumstances than they would have done if they had been alone with the researcher. The respondents may also conveniently conform to the majority’s opinion or, conversely, may rebel against it. In both cases, however, misleading data could be provided.

Prior research studies relevant to this paper are limited (i.e., no study would show the principles of cooperation to reduce aggressive behaviour on the examples of forest animals). This limitation can be considered a challenging opportunity to identify gaps and present further development in the field. This article aimed to compare the effectiveness of methods and approaches to reduce the rate of aggressive manifestations, which is a possible topic for further research via a longitudinal study with a more significant number of probands.

This research is complemented by several studies that have already examined the effect of wilderness therapy or Shinrin-yoku on reducing aggressiveness. So far, no study has combined wilderness therapy, Shinrin-yoku, forest pedagogy, observational learning and the use of projective tests in one piece of research, compared to previous ones. The contribution of this research can be seen in the purposeful connection of the mixed methods approach and testing probands before and after forest therapy by selected projective tests. The paper’s novelty can be found because forest animals’ emotional life has become a mirror with an educational aspect for young people with aggressive behaviour. Science tends to degrade animal emotional manifestations as mere instincts [112], although several scientific works are based on similar animal emotional expressions [113,114], covering only selected partial aspects.

## 5. Conclusions

The importance of this research is evident due to the increasing number of aggressive manifestations of adolescents. This paper aimed to verify the transformation in adolescents ‘attitudes and manners based on a mixed-methods approach and identify whether probands’ teamwork and social adaptation has improved while simultaneously reducing aggressive manifestations. Thanks to the triangulation of methods, we obtained interesting empirical material that would not have been reached by using only one data collection technique. Projective tests provided information about the personality’s internal structure, balance and maturity, perceptual-cognitive level, and reality contact.

The assessment or measurement of aggression is very complex. Projective tests evaluate personality traits, reliably differentiating predispositions to aggressive manifestations rather than aggressive behaviour as such. Projective tests are less transparent, so it is difficult for the tested person to respond deceptively to cover up his/her antisocial and aggressive tendencies. The results suggest that the combination of forest therapy and projective methods appears appropriate and complementary and improves clinical knowledge in assessing aggressive behaviour.

These results can be relevant for policymakers and stakeholders involved in medicine and education to utilise these proposals to design and develop successful strategies and tools to promote this innovative approach. Forest animals showed adolescents ways of communication, cooperation, and adaptability, care for others, i.e., characteristics without which no community can work.

## Figures and Tables

**Table 1:** Group averages of ROR test before and after forest therapy.

ROR		Group A ^1^		Statistical Test Results	Group B ^1^		Statistical Test Results
	Control Group	Before Therapy	After Therapy		Before Therapy	After Therapy	
Pure colour answers	0.41	0.83 ± 0.06	0.79 ± 0.05	^3^ *p* < 0.01 *	0.59 ± 0.13	0.51 ± 0.10	^2^ *p* < 0.01 *
				z = 4.62			t = 4.75
Interpretation of animals	4.99	6.26 ± 0.77	5.87 ± 0.64	^3^ *p* < 0.01 *	6.87 ± 1.14	5.91 ± 0.83	^3^ *p* < 0.01 *
				z = 4.27			z = 4.80
Interpretation of blood	0.04	0.19 ± 0.05	0.11 ± 0.04	^2^ *p* < 0.01 *	0.13 ± 0.06	0.08 ± 0.02	^3^ *p* < 0.01 *
				t = 8.44			z = 4.11
Contact with reality	4.72	5.09 ± 0.88	5.01 ± 0.89	^2^ *p* = 0.01 *	4.72 ± 0.79	3.99 ± 0.87	^3^ *p* < 0.01 *
				t = 2.19			z = 3.62

^1^ Values represent mean values ± standard deviations. ^2^ *T*-test for dependent samples (α = 5%). ^3^ Wilcoxon matched pairs test (α = 5%). Both tests were used to detect statistically significant differences before and after the experimental intervention. The * character means a statistically significant difference.

**Table 2:** Group averages for The Hand Test categories related to the distance from reality and the level of pathology before and after forest therapy.

Hand Test	Control Group	Group A ^1^		Statistical Test Results	Group B ^1^		Statistical Test Results
		Before Therapy	After Therapy		Before Therapy	After Therapy	
Non-adaptive behaviour	0.38	0.51 ± 0.06	0.42 ± 0.08	^2^ *p* < 0.01 *	0.62 ± 0.09	0.48 ± 0.11	^2^ *p* < 0.01 *
				t = 6.35			t = 5.38
Pathological manifestations	0.91	1.39 ± 0.15	1.18 ± 0.09	^2^ *p* < 0.01 *	2.01 ± 0.17	2.01 ± 0.16	^3^ *p* = 0.74
				t = 7.12			z = 0.33

^1^ Values represent the arithmetic mean ± standard deviation. ^2^ *T*-test for dependent samples (α = 5%). ^3^ Wilcoxon matched pairs test (α = 5%). Both tests were used to detect statistically significant differences before and after the experimental intervention. The * character means a statistically significant difference.

## Data Availability

The data presented in this study are available on request from the corresponding author. The data are not publicly available due to further evaluation within the research.
